# Effects of antidepressants on parameters, melondiadehyde, and diphenyl-2-picryl-hydrazyl levels in mice spermatozoa

**Published:** 2018-06

**Authors:** Ladan Bandegi, Morteza Anvari, Mahmood Vakili, Arezoo Khoradmehr, Aghdas Mirjalili, Ali Reza Talebi

**Affiliations:** 1 *Department of Biology and Anatomy, Shahid Sadoughi University of Medical Sciences, Yazd, Iran.*; 2 *Research and Clinical Center for Infertility, Yazd Reproductive Sciences Institute, Shahid Sadoughi University of Medical Sciences, Yazd, Iran. *; 3 *Department of Community Medicine, School of Medicine, Shahid Sadoughi University of Medical Sciences, Yazd, Iran.*

**Keywords:** Amitriptyline, Venlafaxine, MDA, Sperm chromatin

## Abstract

**Background:**

Prescribing antidepressant drugs is becoming common. These drugs are known to affect sexual functions.

**Objective:**

The study is aimed to assess the effects of amitriptyline and venlafaxine on sperm parameters and evaluate Malondialdehyde (MDA) and 1, 1-Diphenyl-2-picryl-hydrazyl values in BALB/ mice spermatozoa.

**Materials and Methods:**

Forty adult male BALB/c mice were separated into five groups. Group Ι (control) received distilled water; group ΙΙ amitriptyline (4 mg/kg); group ΙΙΙ amitriptyline (4 mg/kg) +vitamin C (10 mg/kg); group ΙV venlafaxine (2 mg/kg); and group V received vitamin C (10 mg/kg) + venlafaxine (2 mg/kg). All drugs were administered by oral gavage for 35 days. After excision of caudal epididymis, it was located in 1 mL Ham's F10 medium at 37oC for 15 min and then analysis of sperm parameters was performed. To examine lipid peroxidation and total antioxidant capacity, the MDA and 1, 1-Diphenyl-2-picryl-hydrazyl were measured, respectively.

**Results:**

The mean sperm parameters in the group treated with amitriptyline were significantly lower than in the other groups. MDA tests showed a significant difference between amitriptyline and control groups (p=0.007).

**Conclusion:**

The results of this study showed that amitriptyline consumption can weaken sperm parameters, which can be attributed to the increased production of ROS and toxicity resulting from amitriptyline consumption. Moreover, venlafaxine improved sperm parameters in mice and the lipid peroxidation in this group did not change compared to the control group.

## Introduction

Antidepressant drugs are widely used for the treatment of depression, anxiety-related diseases, and stress (1). The classification of antidepressant drugs is dependent on the drugs chemical structure and how the drugs function (2). Accordingly, antidepressant drugs fall into five families namely tricyclic antidepressants, selective serotonin reuptake inhibitors (SSRIs), serotonin-norepinephrine reuptake inhibitors, monoamine oxidase inhibitors, and atypical antidepressants (3). Reports indicate that antidepressant drugs have a toxic influence on body organs, particularly the male reproductive system. Venlafaxine is one of the drugs belonging to the serotonin-norepinephrine reuptake inhibitor family. Venlafaxine, similar to tricyclic antidepressants, inhibits the reuptake of serotonin and norepinephrine, but the adverse effects are lower (4). The treatment of rats with venlafaxine has indicated that this drug modulates the oxidative stress induced by depression in the brain and medulla of rats (5). 

Amitriptyline is a component of the tricyclic antidepressant family (6). Amitriptyline is used in the treatment of depression, fibromyalgia, chronic fatigue syndrome, migraine, irritable bowel syndrome, and atypical facial pain. Amitriptyline acts by inhibiting the reuptake of serotonin and norepinephrine (6). Several studies have shown that amitriptyline has toxic effects that work through the enhancement of oxidative stress (7). A number of studies have demonstrated a high level of lipid peroxidation in the presence of amitriptyline (7, 8). Oxidative stress occurs as the result of the imbalance between reactive oxygen species (ROS) production and the activity of the antioxidant system (9, 10). The increased production of ROS may cause infertility (11). The targets of ROS in biological systems are nuclear DNA, proteins, and lipids. Owing to the existence of many polyunsaturated fatty acids in the spermatozoa membrane, it is very sensitive to ROS attacks (12). 

Different factors increase testicular oxidative stress, including: exposure to pesticides, industrial chemical materials, and high doses of metals like iron, cadmium and lead, as well as several drugs such as antineoplastic drugs, antibacterials, antimalarials, calcium canal blockers, and antidepressants (10). Malondialdehyde (MDA) is one of the by-products of lipid peroxidation, that are produced by enzymatic or non-enzymatic processes (14). Increased lipid peroxidation and consequently enhanced MDA, may alter sperm functions, thus decreasing male fertility (15). MDA concentration and diphenyl-1-picrylhydrazyl (DPPH) represent lipid peroxidation and the total antioxidant capacity, respectively. In this study, we investigated the effects of amitriptyline and venlafaxine drugs on sperm parameters and the levels of MDA and DPPH of sperm in BALB/c mice. 

Since not much attention has been paid to the effect of antidepressant drugs, especially the SNRL family of drugs, on sperm parameters and lipid peroxidation, the present study aims to examine the impact of amitriptyline and venlafaxine drugs on sperm parameters and on MDA and DPPH levels in BALB/C mice.

## Materials and methods

**Animals and treatments**

In this experimental study, 40 adult male BALB/c mice, weighing 20-25 gr and age of 8 wk were obtained from animal house. The mice were kept for 12 hr in light/dark cycle with 22-25^o^C of temperature, and were fed with standard animal chow and water. 

Venlafaxine and amitriptyline were obtained from Parsdarou and Abidi Pharmaceutical Company, Iran, respectively. Venlafaxine and vitamin C were dissolved in physiological saline and amitriptyline was dissolved distilled water. The animals were divided into five groups. 

The group Ι (controls) was treated with distilled water, group ΙΙ received amitriptyline (4 mg/kg) (2), group ΙΙΙ amitriptyline (4 mg/kg)+vitamin C (10 mg/kg) (2, 16), group ΙV venlafaxine (2 mg/kg) (5), and group V vitamin C (10 mg/kg)+venlafaxine (2 mg/kg) (5, 16), to form the gavage. All groups were treated in mouse spermatozoa period (35 days), and after the treatment period, they were killed by the displacement of the neck vertebrae.

**Epididymal sperm aspiration and sperm analysis**

Thirty-five days later, after the mice were killed and the caudal epididymis was brought out, it was placed in 1 mL of culture medium Hams F10, which has been previously placed in the CO_2_-bearing incubator. By slowly tearing up the tissue, sperms were extracted and placed in an incubator with 5% CO_2_ at 37^o^C for 20 min. To analyze the sperms, viability was evaluated by stained eosin, and then motility and sperm concentration were evaluated via Makler chamber with ×100 magnification optical microscopes (Olympus Co. Tokyo, Japan). 

Motility was classified into progressive (grade a and b: fast and slow) and non-progressive (grade c) spermatozoa. For evaluating normal morphology, stained Papanicolaou was used and 200 spermatozoa were counted using a ×100 magnification light microscope (17). All the chemical agents and pigments were purchased from Merck & Co, Germany.

**Lipid peroxidation**

The concentration of the lipid peroxidation product (MDA) or the thiobarbituric acid-reactive substance was determined according to the modified method of Uchiyama and Mihara. In brief, 0.375 ml HCL 20% and 0.375 ml TBA 0.6% were added to 0.1 ml semen plasma. The mixture was heated for 60 min in a boiling water bath. After cooling, 1.25 ml of n-butanol was added, and the mixture was shaken. Thereafter, the mixture was centrifuged at 2000×g for 5 min, and the absorbance of the upper solution was determined at 532 nm (18).

**DPPH measurement**

The total antioxidant capacity was performed through the trapping of plasma-free radicals by reducing diphenyl-2-picrylhydrazyl. The DPPH stocks were prepared in methanol, which was buffered by acetic acid. The buffered methanol was prepared by mixing 40 mL of acetate buffer 0.1 M to 60 mL of methanol. The other solutions and chemicals were analytic grade. The reaction tubes were wrapped in aluminum foils and kept at 30ºC for 30 min. All measurements were performed at weak light at the wavelength of 517 nm using a spectrophotometer (19).

**Ethical consideration**

The experimental project was confirmed by the ethics committee of Shahid Sadoughi University of Medical Sciences, Yazd, Iran (IR.SSU.MEDICINE.REC.1394.274). The protocols of the Institutional Animal Care and Use Committee were followed during the course of the experiment in terms of the handling, maintenance, treatment, and killing of the animals.

**Statistical analysis**

All data were analyzed by SPSS software (Statistical Package for the Social Sciences, version 18.0, SPSS Inc., Chicago, Illinois, USA). One-way ANOVA was applied to evaluate the data, and LSD post-test was performed to determine the difference between the two groups. Two-sided p<0.05 indicated a statistically significant difference between sperm evaluations.

## Results

**Sperm parameters**

Table Ι indicates that in all sperm parameters including progressive motility, non-progressive motility, immotile, normal morphology, viability except concentration, there was a significant difference between groups ΙΙ and ΙV )p=0.001, 0.007, 0.001, 0.005, and 0.001 respectively). Moreover, the motility parameters, viability and immotile spermatozoa were significantly different between groups ΙΙ and ΙΙΙ (p=0.009, 0.001, and 0.001 respectively). 

There was a significant difference between groups Ι and ΙV in normal morphology, non-progressive motility, and viability (p=0.024, 0.038 and 0.019 respectively). On the other hand, there was a significant difference between groups Ι and ΙΙ with regard to progressive motility, immotile, sperm concentration and viability parameters (p=0.001, 0.001, 0.046, and 0.001 respectively).

**Assessment of Oxidative Stress**

According to Table II, the amount of MDA in group ΙΙ is higher than in the other groups, but it is lower in group V than in the other groups. The amount of MDA in drug groups with vitamin C is lower than the drug groups without vitamin C. However, there was a significant difference between groups ΙV and V (p=0.002), groups Ι and ΙΙ (p=0.007) and was not significant between groups ΙΙ and ΙΙΙ. In the DPPH test, the total antioxidant capacity amount in groups ΙΙ, ΙΙΙ, and ΙV decreased compared to the control group, but it increased in group V compared to the other groups. However, these results were not statistically significant.

**Table I:** The results of sperm parameter analysis in study groups (n=8)

**Variable**	**Group Ι**	**Group ΙΙ**	**Group ΙΙΙ**	**Group ΙV**	**Group V**	**p-value**
Sperm Concentration (10^6^)	22.41 ± 4.38	18.11 ± 3.72	16.86 ± 3.15	22.06 ± 2.45	22.61 ± 3.09	0.046^a^ 0.011^b^
Progressive Motility (%)	44.12 ± 22.50	13.75 ± 4.49	30.87 ± 6.53	52.75 ± 5.39	48.50 ± 7.23	<0.001^a^ 0.04^b^ 0.009^e^ <0.001^g^
Non-progressive motility (%)	16.25 ± 10.84	13.37 ± 5.57	22.75 ± 8.94	25.50 ± 10.33	30.25 ± 7.42	0.038^c^ 0.002^d^ 0.035^e^ 0.007^g^
Immotile (%)	39.12 ± 23.35	71.75 ± 5.57	46.75 ± 9.91	21.25 ± 12.17	21.12 ± 4.91	<0.001^a^ 0.012^c^ 0.012^d^ 0.001^e^ <0.001^g^
Normal morphology (%)	43.71 ± 15.39	38.83 ± 11.39	47.00 ± 10.03	58.5 ± 10.47	54.37 ± 13.67	0.024^c^ 0.005^g^
Viability (%)	64.62 ± 22.58	31.25 ± 6.88	54.12 ± 10.10	80.25 ± 12.42	80.62 ± 5.15	<0.001^a^ 0.019^c^ 0.017^d^ 0.001^e^ <0.001^g^

To analyze data we used standard s one way ANOVA test.

The mean difference was significant at the 0.05 level. All data are presented as mean ± SD)

a : Significant difference between group Ι and ΙΙ

b : Significant difference between group Ι and ΙΙΙ

c : Significant difference between group Ι and ΙV

d : Significant difference between group Ι and V

e : Significant difference between group ΙΙ and ΙΙΙ

f : Significant difference between group ΙV and V

g : Significant difference between group ΙΙ and ΙV

**Table II:** Seminal plasma levels of total antioxidant capacity and malondialdehyde in study groups (n=8)

**Variable**	**Group Ι **	**Group ΙΙ**	**Group ΙΙΙ**	**Group ΙV**	**Group V**	**p-value**
MDA (nmol/ml)	0.0075 ± 0.002	0.0111 ± 0.003	0.0085 ± 0.002	0.0101 ± 0.002	0.0059 ± 0.0009	0.007^a^ 0.002^b^
DPPH (µM)	991.92 ± 124.53	785.71 ± 58.961	838.40 ± 60.58	873.18 ± 71.14	935.65 ± 46.36	

To analyze data we used standard s one way ANOVA test. The mean difference was significant at the 0.05 level. All data are presented as mean ± SD

MDA: Malondialdehyde

DPPH: Diphenyl-1-picrylhy drazyl

a : Significant difference between group Ι and ΙΙ

b : Significant difference between group ΙV and V

## Discussion

The mains finding of the present study shows that amitriptyline consumption can weaken sperm parameters. Moreover, venlafaxine can improve sperm parameters in mice.

Different studies indicated that antidepressant drugs could have a negative effect on the male reproductive system. However, there is little knowledge of the effect of antidepressant drugs consumption on the ROS production. The purpose of this study is to investigate the influence of antidepressant drugs on sperms parameters and ROS production. In addition, the influence of ROS production on the sperms parameters has been investigated. Our finding showed the toxicity of amitriptyline lead to decrease in the sperm parameters including sperm concentration, progressive motility, and viability. In our study, amitriptyline alone and with vitamin c showed a significant difference in sperm parameters. Actually, consumption of amitriptyline with vitamin c could improve progressive motility, non-progressive motility, viability and decrease immotile. In agreement with our study, Hassanane and co-workers showed a decrease in sperm concentration as well as an increase in morphological abnormalities in the sperm of amitriptyline-treated mice (2). 

It has been reported in the literature that the consumption 75-300 mg of trimipramin for 8 wks affects spermatogenesis which is in agreement with our results from amitriptyline-treated mice (20). The finding of the present study showed that venlafaxine than control group increased non-progressive, normal morphology, viability and decreased immotile.

Also, no significant change was observed in sperm concentration. Tanrikut and colleagues have analyzed the results of male infertility examination on two men who were receiving the citalopram, sertraline, and venlafaxine. The semen analysis of patient 1 who was receiving citalopram indicated oligospermia and 1% motility.

The semen analysis of patient 2 who was receiving sertraline revealed normal volume and no motility sperm (21). In another study conducted by Tanrikut, the consumption of paroxetine and SSRI for 5 wks had no adverse effects on key sperm parameters, However, it increased the DNA fragmentation significantly (22). The effects of SSRIs on sperm parameters have been investigated in two separate studies and it has been reported that SSRI exerted a negative influence on sperm parameters and quality, which is in contrast with our results from venlafaxine and venlafaxine-vitamin C groups (23, 24). In a similar series of experiments on patients who consumed SSRI for six months, Safarinejad observed a decrease in sperm parameters, a significant increase in DNA damage (25). These discrepancies between our results and the results of previous studies can be due to the difference in types of drugs and their dosage which have been used and the fact that the current study was performed on mice. In another study, Atli and colleagues showed that using sertraline decreases the sperm concentration and morphology. In addition, it causes a decrease in GSH level, increase in MDA and changes the level of serum LH and testosterone (26).

Although the exact mechanism of the effects of antidepressant drugs on the reproductive system has not yet been understood well, it has been suggested that neuroendocrine factors may be involved in this mechanism (27). It has been shown that increase of serotonin in humans can affect ejaculation and sperm transition. For example, serotonin causes the inhibition of dopamine by increasing the prolactin level and stimulating the activity of prolactin-releasing factors (27). Increase in prolactin, causes the spermatogenesis to suppresses the defective sperm motility and changes the sperm quality (28). 

Furthermore, Weydt and colleagues, in 2011, reported a few cases of gynecomastia due to the consumption of venlafaxine and fluoxetine, which were all associated with increased prolactin levels (29). In another study, SSRIs and SNRIs declined dopamine neurotransmissions with hyperprolactinemia (30). It is known that serotonergic antidepressants can potentiate 5Hydroxytryptophan receptor-mediated stimulation of prolactin secretion (31). It is unclear that how antidepressants affect spermatogenesis or sperm motility, however, it has been suggested that it may have an influence on sperm PH or viscosity, on nitric oxide concentration, or physiologic regulator of sperm motility (20).

The high levels of ROS affect the normal sperm quality (mobility, viability, function) by interacting with lipid membrane, proteins, nuclear and mitochondria DNA. Therefore, in our study to examine the production of ROS by drugs and their effects on the lipid peroxidation and also to evaluate total capacity of antioxidant, the amounts of MDA and DPPH were measured respectively. 

Our results showed that the seminal plasma DPPH, showed no significant change. However, the result of MDA test between group Ι and ΙΙ, indicated a significant increase. These results are in accordance with Taziki and colleagues that showed amitriptyline led to a significant increase in lipid peroxide in the hepatocytes of rats and the amount of thiobarbituric acid-reactive substance (32). In 2010, Afify and colleagues reported that amitriptyline in high dose led to liver cytotoxicity and reduced testicular function (6).

Cordero and co-workers reported the mitochondrial cytotoxicity in patients who used amitriptyline (8). In this study, using amitriptyline by depressed patients, leads to increase in lipid peroxidation and reduction in coenzyme Q 10 (membrane antioxidant) and citrate synthesis activity (8). Our finding showed that MDA test between group Ι and ΙV was not significant but indicated a significant alteration between group ΙV and V. In other words, the vitamin c consumption in group V could decrease the MDA level compared to group ΙV.

According to Abdel-Wahab, venlafaxine is an effective antioxidant against lipid peroxidation damage and increases antioxidant defense line. Also in this study, the amount of MDA decreased in the depression group that was under treatment with venlafaxine (18). The level of lipid peroxidation in brain cortex, medulla, and erythrocyte was lower in the venlafaxine group compared to the depressed group in Eren’s study which can be a confirmation to our results (5). El-Din and colleagues examined the influence of duloxetine on the biochemical and immunocytochemical alteration in the testes of rats, and reported increases in MDA level and decreases in testosterone, LH and FSH levels (33). 

Also, in our study observed that MDA level in group V than in group ΙV, was a significant decline. By considering the lower level of MDA in group V than in group ΙV, it can be concluded that supplemented ascorbic acid in group V improved the effects of lipid peroxidation caused by venlafaxine. In accordance with our results, Ayinde and colleagues reported that daily consumption of supplemented ascorbic acid can decrease stress oxidative and alteration spermatogenesis in albino rats (34). We observed that seminal plasma lipid peroxidation reduced in the venlafaxine group compared to the amitriptyline group; however, no significant change was observed between these two groups. 

In the current study, the use of venlafaxine along with supplemental ascorbic acid significantly decreased lipid peroxidation. Moreover, considering the results of lipid peroxidation and of similar studies done so far on the two drugs, it seems that venlafaxine has less influence on male fertility than amitriptyline due to less ROS production and less influence of drugs on sperm parameters. This study shows that in the amitriptyline-vitamin C-treated mice, the sperm motility is improved and immotile is decreased, while in the venlafaxine-vitamin C-treated mice sperm viability is improved. Ascorbic acid plays an important role in the distinction process of spermatogenesis. The consumption of antioxidants causes conflicts with ROS. ROS might destroy testicular germ cells which leads to a decrease in sperm concentration and an increase in abnormal sperm (35). It should also be considered that with increases in lipid peroxidation the sperm motility is likely to be low (36). 

Griveau and colleagues showed that ROS increases the lipid peroxidation which causes a decline in sperm mobility and then a reduction in unsaturated fatty acids (37). In accordance with Griveau’s results, sperm concentration in the amitriptyline group decreased, which can be attributed to the high production of ROS in this group. In our study, a comparison between the amitriptyline and venlafaxine groups showed that lipid peroxidation has been increased, but it was not statistically significant. Simultaneous consumption of supplemental ascorbic acid and amitriptyline decreased the lipid peroxidation; however, it did not result in any significant change between the two groups. These findings further support the idea of Das who reported a negative correlation between ascorbic acid and MDA relative to the defensive role of ascorbic acid against the process of lipid peroxidation (38). 

Also, in this study, there was a significant positive correlation between the seminal plasma of ascorbic acid and the concentration, motility, and normal morphology of sperm which are in accordance with the results of Thiele (39). Hence, it is recommended for patients who are suffering from depression and are willing to have offspring to use antioxidants along with antidepressants drugs to reduce the effects of oxidative stress induced by drug consumption.

## Conclusion

Our findings show that the administration of amitriptyline in mice has more detrimental effects on sperm parameters and causes more peroxidation than venlafaxine dose.

## Note

***This article extracted from M.Sc. thesis. (Ladan Bandegi)***
